# Negative expression of N-acetylglucosaminyltransferase V in oral squamous cell carcinoma correlates with poor prognosis

**DOI:** 10.1186/2193-1801-2-657

**Published:** 2013-12-06

**Authors:** Kahori Seto, Fumihiko Uchida, Osamu Baba, Masanobu Yamatoji, Rei Karube, Eiji Warabi, Satoshi Sakai, Shogo Hasegawa, Kenji Yamagata, Toru Yanagawa, Kojiro Onizawa, Eiji Miyoshi, Junichi Shoda, Hiroki Bukawa

**Affiliations:** Oral and Maxillofacial Surgery, Clinical Sciences, Graduate School of Comprehensive Human Sciences, University of Tsukuba, 1-1-1 Tennodai, Tsukuba, Ibaraki, Japan; Department of Oral and Maxillofacial Surgery, Faculty of Medicine, University of Tsukuba, 1-1-1 Tennodai, Tsukuba, Ibaraki, Japan; Medical Science Section, Faculty of Medicine, University of Tsukuba Graduate School, 1-1-1 Tennodai, Tsukuba, Ibaraki, Japan; Department of Oral and Maxillofacial Surgery, Mito Kyodo General Hospital, Tsukuba University Hospital, Mito Medical Center, 3-2-7 Miya, Mito, Ibaraki, Japan; Cardiovascular Division, Faculty of Medicine, University of Tsukuba, 1-1-1 Tennodai, Tsukuba, Ibaraki, Japan; Molecular Biochemistry and Clinical Investigation, Osaka University Graduate School of Medicine, 1-7, Yamadaoka, Suita, Osaka, Japan; Department of Gastroenterology, Faculty of Medicine, University of Tsukuba, 1-1-1 Tennodai, Tsukuba, Ibaraki, Japan

**Keywords:** N-acetylglucosaminyltransferase V, GnT-V, Oral squamous cell carcinoma, OSCC, Biomarker

## Abstract

N-acetylglucosaminyltransferase V (GnT-V), an enzyme with a key role in the branching of asparagine-linked oligosaccharides, is strongly linked to tumor invasion and metastasis of many solid tumors. Here we searched for correlations between the clinical features of patients with oral squamous cell carcinoma (OSCC) and GnT-V expression in the tumor, and we studied the feasibility of using GnT-V as a marker for oral cancer prognosis. Samples from 68 patients with OSCC were examined by immunohistochemistry using antibodies against GnT-V. Correlations between the expression level of GnT-V in the tumor and patient clinical features were statistically analyzed. Positive GnT-V expression was found in 48 cases (70.6%), and negative GnT-V expression was found in 20 cases (29.4%). Negative GnT-V expression was associated with mode of invasion by multiple logistic regression analysis (OR: 3.605; P = 0.048). Biological characteristics of tumors and the Ki-67 labeling index were higher in tumors with negative GnT-V expression than in those with positive GnT-V expression, although the difference was not significant (P = 0.176). Patients with negative GnT-V expression had significantly shorter survival than those with tumors having positive GnT-V expression (5-year survival rate, 58.2% and 86.5%, respectively; P = 0.025). Negative GnT-V expression was a significant unfavorable prognostic factor for OSCC (hazard ratio, 4.246; P = 0.045). The loss of GnT-V expression is a likely indicator of tumors with high potential of tumor invasion and poor prognosis in OSCC patients.

## Introduction

Head and neck carcinoma, which includes cancers of the oral cavity, oropharynx, larynx, and hypopharynx, is the sixth most common cancer worldwide and has an incidence of around 600,000 cases per year (Kamangar et al. 2006). Oral cancer, the largest subset of head and neck cancer, has become one of the most lethal malignancies (Chen et al. 2013), of which oral squamous cell carcinoma (OSCC) is the most frequent histological type (Parkin et al. 2005). The current management and treatment of OSCC involves multimodal approaches comprising surgery, chemotherapy, and radiotherapy (Seiwert and Cohen 2005). Despite recent advances in early detection, diagnosis, and treatment, the 5-year survival for patients with OSCC has remained at 50% for the past 30 years (Forastiere et al. 2003). Because of the high prevalence and mortality rate of oral cancers, prevention and early intervention are important strategies for managing the disease.

Glycosylation of cell-surface glycoproteins is widely accepted to play a key role in various specific biological interactions. The glycosyltransferase plays a crucial role on the protein glycosylation. Glycosyltransferase, located in the Golgi apparatus, includes at least six N-acetylglucosaminyltransferase (GnT) defined as GnT-I-VI (Taniguchi et al. 1999). GnT-V, a glycosyltransferase encoded by the *Mgat5* gene that catalyzes the formation of β1,6GlcNAc (N-acetylglucosamine) branches on N-glycans, is believed to be associated with cancer growth and metastasis (Taniguchi et al. 1999; Lau and Dennis 2008). Moreover GnT-V protein could results in tumor angiogenesis, and its mechanism as an inducer of angiogenesis was different from original function as a glycosyltransferase (Saito et al. 2002).

Numerous studies have shown that GnT-V is positively correlated with malignancy in many types of tumor, including breast, colon, endometrial, and ovarian mucinous tumors (Fernandes et al. 1991; Murata et al. 2000; Yamamoto et al. 2007; Takahashi et al. 2009). In contrast, the opposite results have been found for lung, thyroid, and liver tumors. As such, GnT-V expression and its functional and prognostic significance in human cancer remain controversial. The relationship between GnT-V expression and malignancy has been studied in many types of tumor, but not in human oral SCC. In vitro analysis, it was reported that the decrease inβ1, 6GlcNAc branching on cisplatin-resistant human SCC cell line, so the GnT-V expression in SCC may be inversely corelated with prognosis (Nakahara et al. 2003).

In this study, we examined GnT-V expression by immunohistochemistry for surgically resected OSCC and analyzed the correlation with clinical features of OSCC.

## Materials and methods

### Patients and tissue specimens

Tumor specimens were obtained from 68 patients with OSCC seen at the Department of Oral and Maxillofacial Surgery, University of Tsukuba Hospital, Ibaraki, Japan during the period 1994–2004. Patients were followed for more than 60 months. Tumors were staged according to the International Union Against Cancer scheme (Sobin and Wittekind 1997), and clinical data were obtained from patient medical records. Specimens were obtained after patients gave informed consent, and the study protocol was reviewed and approved by the Research Ethics Committee of the University of Tsukuba (H25-43).

### Immunohistochemistry

**Figure 1:**
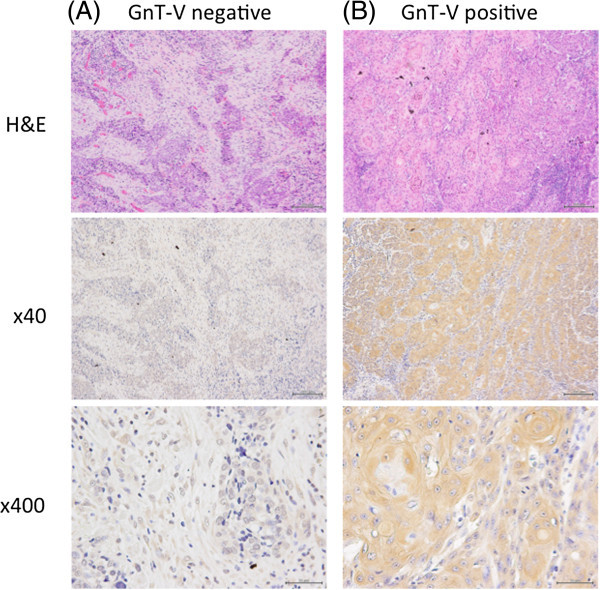
**Representative photomicrographs of immunohistochemical staining with N** ***-*** **acetylglucosaminyltransferase (GnT-V) antibodies Negative staining (total absence of GnT-V immunostaining) (A), and positive staining (B) (original magnification, ×40 and × 400).**

For immunostaining of GnT-V, 2-μm thick sections from patient samples were stained using the Vecta staining kit according to the manufacturer’s instructions with anti-GnT-V antibody obtained from Dr. Eiji Miyoshi (Osaka University, Osaka, Japan). GnT-V expression levels were classified into two groups according to the percentage of positively stained cells in the cancerous area: ≥ 30% (positive) and < 30% (negative) (Takahashi et al. 2009) (Figure 1). The scoring procedure was carried out twice by two independent observers who were blinded to the clinical data.

### Statistical analysis

To simplify the correlation analysis of GnT-V expression with clinical features, tumors were divided into the T-category groups T1 + T2 or T3 + T4. Clinical stage was classified as I + II or III + IV, and differentiation as well-/moderately or poorly differentiated. Anneroth grade to denote tumor invasion was assigned as 1–3 or 4. For univariate analysis, we used the Chi-squared test, Student’s test, or Welch’s *t*-test. For multivariate analysis, we used multiple logistic regression analysis. All analysis was performed using the statistical software package SPSS.

## Results

### Univariate analysis of GnT-V expression

Positive GnT-V expression was observed in 48 specimens (70.6%) and negative GnT-V expression in 20 specimens (29.4%). Table 1 shows the correlation between GnT-V expression and clinicopathological features. The GnT-V-negative group included significantly more young patients (P = 0.006), more males than females (P = 0.028), alcohol consumption (P = 0.027), more invasive tumors (P = 0.016), and a higher 5-year survival rate (P = 0.015). No significant difference in GnT-V expression was observed with respect to other factors, including, smoking, T-category, clinical stage, cellular differentiation, pN positive or negative, local recurrence, lymph node metastasis, and treatment type. No difference was observed between GnT-V expression and p53 expression. Ki-67 labeling index values were higher in tumors with negative GnT-V expression than in those with positive GnT-V expression, but not significantly (P = 0.176) (Table 2).

**Table 1:** **Relationship between GnT-V expression and clinical and clinicopathological characteristics in all 68 patients**

	GnT-V positive	GnT-V negative	P
**Age**			
Average	68.88	59.15	0.006
**Gender**			
Male	22	15	
Female	26	5	0.028
**Alcohol**			
no	33	8	
yes	15	12	0.027
**Smoking**			
no	32	9	
yes	16	11	0.096
**T-Category**			
1, 2	30	13	
3, 4	18	7	0.845
**Clinical Stage**			
I, II	27	9	
III, IV	21	11	0.397
**Differentiation**			
Well	33	9	
Moderate, poor	15	11	0.066
**Mode of invasion**			
1 to 3	32	7	
4	16	13	0.016
**pN**			
Negative	42	15	
Positive	6	5	0.202
**Recurrence**			
Negative	38	15	
Positive	10	5	0.178
**Metastasis**			
Negative	38	14	
Positive	10	6	0.416
**Survival**			
Alive	43	13	
Dead	5	7	0.015
**theraphy**			
Operation	34	11	
Chemoradiotheraphy	14	9	0.208

**Table 2:** **The relationship between GnT-V expression and p53, ki 67 expression**

	GnT-V positive	GnT-V negative	P
**p53**			
Negative	33	16	
Positive	15	4	0.346
**Ki67 mib index (Mean ± SD)**			
	14.0 ± 1.9	19.8 ± 4.6	0.176

### Multivariate analysis of GnT-V expression

The predictor variables in the 68 patients were used in a logistic regression model with GnT-V expression as the dependent variable. The logistic model was constructed using clinical variables, including age, gender, alcohol consumption, smoking, stage, differentiation, and Mode of invasion. Adjusted odds ratios (OR) and P values are shown in Table 3. Negative staining for GnT-V (OR = 3.605 and P = 0.048) was significantly associated with invasion but not with the other variables.

**Table 3:** **Multiple logistic regression analysis for the correlation between GnT-V expression and clinical characteristics**

Characteristics	Odds ratio	P
**Age**	1.048	0.052
**Gender (male/female)**	0.502	0.415
**Alcohol (no alcohol/alcoho drinker)**	1.750	0.464
**Smoking (non smoker/smoker)**	1.378	0.639
**Stage (I + II/III + IV)**	1.135	0.866
**Differentiation (well/moderate + poor)**	1.391	0.597
**Mode of invasion (1-3/4)**	3.605	0.048

### GnT-V expression and prognosis of OSCC

**Figure 2:**
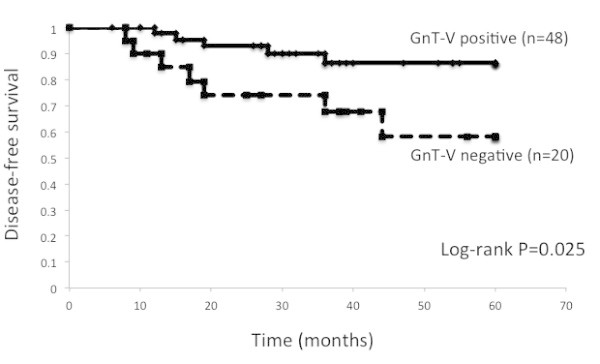
**Kaplan Meier disease-free survival of GnT-V expression in all patients.** The 5-year disease-free survival (DFS) rates of positive GnT-V expression and negative GnT-V expression were 86.5 % and 58.2%, respectively (P = 0.025).

We next analyzed the relationship between GnT-V expression and patient survival and the importance of GnT-V as a prognostic factor. Kaplan-Meier survival curves clearly demonstrated that patients with negative GnT-V expression had significantly shorter survival than patients with positive GnT-V expression (5-year survival rate, 58.2% and 86.5%, respectively; P = 0.025; Figure 2). Cox proportional-hazard analysis was performed to compare the impact of GnT-V expression on survival with currently used clinicopathological prognostic factors such as age, gender, alcohol consumption, smoking consumption, stage, differentiation, and GnT-V expression. Negative GnT-V expression was the only significant unfavorable prognostic factor in our analysis (hazard ratio, 4.246; P = 0.045) (Table 4).

**Table 4:** **Cox proportional hazards model analysis of prognostic factors in patients**

Characteristics	Hazard ratio	95% CI	P
**Age**	1.060	0.992-1.132	0.083
**Gender (male/female)**	0.488	0.093-2.565	0.397
**Alcohol (no alcohol/alcoho drinkerl)**	1.436	0.333-6.199	0.627
**Smoking (non smoker/amoker)**	1.814	0.444-7.408	0.407
**Clinical stage (I, II / III, IV)**	2.428	0.541-10.896	0.247
**Differentiation (well/moderate + poor)**	1.016	0.286-3.612	0.980
**GnT-V expression**	4.246	1.0320-17.586	0.045

## Discussion

Glycosylation is one of the most common posttranslational protein modifications, and nearly half of all known proteins in eukaryotes are glycosylated (Saxon and Bertozzi 2001). Cell surface glycosylation not only regulates the stability and activity of structural proteins and receptors on the cell membrane, but also participates in the maintenance of cell morphology and cell-cell interactions (Hirai-Fujita et al. 2008; Krishnan et al. 2005; Rak et al. 1991). Changes in glycans are associated with many physiological and pathological events, including cell adhesion, migration, and invasion (Dennis et al. 1987).

The present report shows that GnT-V expression in OSCC is associated with age (P = 0.006), gender (P = 0.028), alcohol consumption (P = 0.027), mode of invasion (P = 0.016), and 5-year survival (P = 0.015). Although our results revealed that there were no significant differences between GnT-V expression and T or Clinical stages, it was reported that GnT-V expression is upregulated in the early stages of almost all cancers (Miyoshi et al. 2012). However Multiple logistic regression analysis to determine the correlation between GnT-V expression and clinical and clinicopathological characteristics showed that the cases of negative GnT-V expression tended to be more invasive as determined by Anneroth grade.

Kaplan-Meier survival curves clearly demonstrated that patients with negative GnT-V expression had significantly shorter survival than patients with positive GnT-V expression (5-year survival rate, 58.2% and 86.5%, respectively; P = 0.025; Figure 2). Historogy was significantly correlated with GnT-V expression and low GnT-V expression was more frequently found in squamous cell carcinomas than non-squamous cell carcinomas (Akita 2004). Our data strongly suggested that the relationship between GnT-V expression and the prognosis depends on the histrogical type, as well as the original organ of the cancer. When considering survival rate, the type of treatment (surgery or chemoradiotherapy) was taken into account, but we found no significant correlation between GnT-V expression and treatment type. Moreover, in patients with negative GnT-V expression that correlated with survival rate, we found no significant correlation between GnT-V expression and local recurrence or node metastasis. This suggests that negative GnT-V expression reduces the efficacy of chemoradiotherapy as a second treatment. This implies that OSCC patients with negative GnT-V expression are more likely to have poor prognosis.

The relationship between cisplatin-resistance and α5β1 integrin with β1-6GlcNAc branching has been reported in an established cisplatin-resistant head and neck carcinoma cell line, but reasons for the relationship are unclear (Nakahara et al. 2003). Down-regulation of GnT-V enhances nasopharyngeal carcinoma cell radiosensitivity both in vitro and in vivo, and is linked to the G2-M cell cycle arrest and the reduction of the BcL-2/Bax ratio (Zhuo et al. 2012). Conversely, a correlation was found between the high expression levels of GnT-V in neuroblastoma patients with a favorable prognosis, suggesting that GnT-V can cause neuroblastomas to regress by increasing their susceptibility to apoptosis (Inamori et al. 2006).

Low expression of GnT-V may contribute to altered biological properties of bladder cancer as well as non-small cell lung cancer and hepatocellular carcinoma by decreasing the synthesis of β1-6 branching oligosaccharides of certain target glycoproteins, resulting in shorter survival in patients having tumors with low GnT-V expression compared with patients having tumors with high GnT-V expression (Akita 2004; Ishimura et al. 2006; Ito et al. 2001). The importance of this oligosaccharide structure as a precursor to malignancy differs between organs, and the target substrate of GnT-V might differ between oral cancer and other carcinomas. However, from a clinical background, there is not a significant difference with the tumor differentiation with GnT-V expression in OSCC as observed in the other cancer that prognosis was inversely correlated with GnT-V expression. In addition, since expression of GnT-V expression is low in young people, histologic pattern might be different in GnT-V positive and negative cases.

Taken together, immunohistochemistry of OSCC specimens can provide information that could help physicians make appropriate decisions for the treatment of cancer patients. For example, if GnT-V expression is absent, the tumor is more likely to have poor prognosis, and radical treatment in such a case would be a better choice. However the potential oncogenic role and underling mechanisms of GnT-V in OSCC have not been investigated. Clealy, further studies are needed to elucidate the mechanisms of GnT-V promoting the development and metastasis of OSCC in detail.
